# 4-Chloro-6,7-dimeth­oxy­quinoline

**DOI:** 10.1107/S1600536811042589

**Published:** 2011-10-22

**Authors:** Min Wu

**Affiliations:** a School of Chemistry and Chemical Engineering, Southeast University, Nanjing 211189, People’s Republic of China

## Abstract

The title mol­ecule, C_11_H_10_ClNO_2_, is almost planar with the C atoms of the meth­oxy groups deviating by −0.082 (2) and 0.020 (2) Å from the least-squares plane defined by the atoms of the quinoline ring system (r.m.s. deviation = 0.002 Å). An intra­molecular C—H⋯Cl inter­action generates an *S*(5) ring motif.

## Related literature

For related structures, see: Davies & Bond (2001 ▶); Yathirajan *et al.* (2007 ▶). For biological properties of quinoline derivatives, see: Franck *et al.* (2004 ▶); Moret *et al.* (2006 ▶); Furuta *et al.* (2006 ▶); Ilovich *et al.* (2008 ▶). For hydrogen-bond motifs, see: Bernstein *et al.* (1995 ▶).
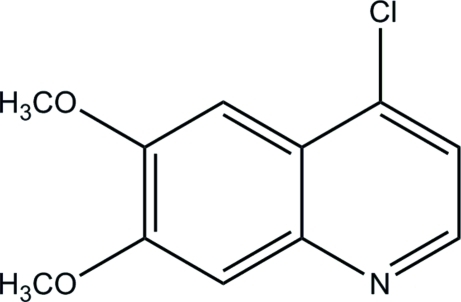


## Experimental

### 

#### Crystal data

C_11_H_10_ClNO_2_
*M* *_r_* = 223.65
Monoclinic, 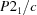

*a* = 12.5530 (17) Å
*b* = 4.6499 (7) Å
*c* = 18.274 (3) Å
β = 105.786 (2)°
*V* = 1026.4 (3) Å^3^
*Z* = 4
Mo *K*α radiation
μ = 0.35 mm^−1^
*T* = 296 K
0.3 × 0.2 × 0.2 mm

#### Data collection

Rigaku SCXmini diffractometer
6840 measured reflections
1808 independent reflections
1542 reflections with *I* > 2σ(*I*)
*R*_int_ = 0.034
3 standard reflections every 150 reflections  intensity decay: none

#### Refinement

*R*[*F* ^2^ > 2σ(*F* ^2^)] = 0.037
*wR*(*F* ^2^) = 0.115
*S* = 1.08
1808 reflections
139 parameters
1 restraint
H-atom parameters constrained
Δρ_max_ = 0.29 e Å^−3^
Δρ_min_ = −0.22 e Å^−3^

### 

Data collection: *CrystalClear* (Rigaku, 2005 ▶); cell refinement: *CrystalClear*; data reduction: *CrystalClear*; program(s) used to solve structure: *SHELXS97* (Sheldrick, 2008 ▶); program(s) used to refine structure: *SHELXL97* (Sheldrick, 2008 ▶); molecular graphics: *SHELXTL* (Sheldrick, 2008 ▶); software used to prepare material for publication: *SHELXTL*.

## Supplementary Material

Crystal structure: contains datablock(s) I, global. DOI: 10.1107/S1600536811042589/lr2029sup1.cif

Structure factors: contains datablock(s) I. DOI: 10.1107/S1600536811042589/lr2029Isup3.hkl

Supplementary material file. DOI: 10.1107/S1600536811042589/lr2029Isup3.cml

Additional supplementary materials:  crystallographic information; 3D view; checkCIF report

## Figures and Tables

**Table 1:** Hydrogen-bond geometry (Å, °)

*D*—H⋯*A*	*D*—H	H⋯*A*	*D*⋯*A*	*D*—H⋯*A*
C8—H8⋯Cl1	0.93	2.70	3.0827 (17)	105

**Fig. 1.**
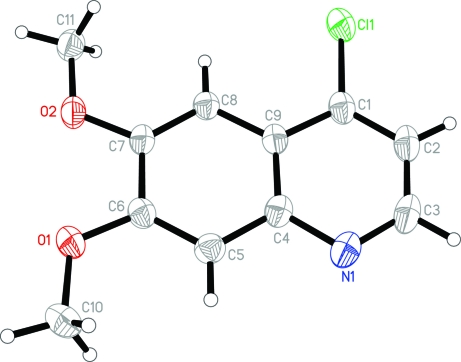
The molecular structure of the title compound, displacement ellipsoids are drawn at the 30% probability level.

**Fig. 2.**
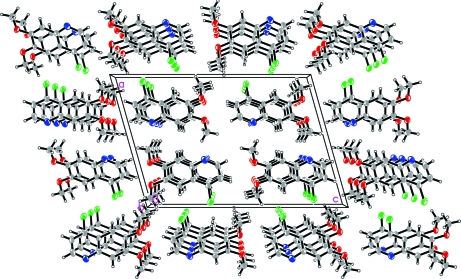
Partial packing view of the title compound, viewed down the b axis.

## supplementary crystallographic information

### Comment

Quinoline derivatives have been interesting to researchers for many years because a large number of natural products contain these heterocycles and also because their varied biological activities ( Franck *et al.* 2004; Moret *et al.* 2006; Furuta *et al.* 2006 & Ilovich *et al.* 2008).

Prompted by the properties of quinoline derivatives, the title compound, C_11_H_10_ClNO_2_, has been synthesized. Bond lengths and angles are in the usual range (Davies & Bond 2001 & Yathirajan *et al.* 2007) and the whole molecule is almost planar with the carbon atoms of the methoxyl groups deviating 0.08 (C10) and 0.02Å (C11) from the least-square plane defined by the atoms of the quinoline ring. There are intramolecular C8 — H8 ···Cl1 interactions (Table 1) generating S(5) ring motifs (Bernstein *et al.* 1995) . Figure 1 shows the molecular structure of the title compound and Figure 2 is a partial paking view of the crystal structure down the *b* axis.

### Experimental

A mixture of 6,7- dimethoxynaphthalen-1-ol (20.4 g, 100 mmol) and POCl_3_ (60 ml, 640 mmol) was heated under reflux for 6 h. The excess of phosporus oxychoride was distilled out under reduced pressure. 200 g crush ice was added to the residue followed by 50% aqueous NaOH until the pH was adjusted to 8. The resulting solid was collected by filtration and washed with water to give the crude product. Purification of the crude product by a column chromatography (petroleum ether: EtOAc = 8:1 v.v) afforded the title compound (15.6 g, 70%) as pink crystals.The purity of the product, 4- chloro- 6, 7- dimethoxy- quinoline, was determined using a reversed- phase C-18 analytical HPLC column (99% purity). Crystals of the title compound suitable for *X*– ray diffraction were obtained by slow evaporation of methanol solution at room temperature. m. p. 403- 404 K; ^1^H NMR (DMSO– d_6_): δ8.57 (d, J = 5.1 Hz, 1H), 7.40 (d, J = 4.8 Hz, 1H), 7.37 (s, 1H), 7.32 (s, 1H), 4.04 (s, 3H), 4.03 (s. 3H). MS (ESI, *m/z*): 224 (*M*+1).

### Refinement

All H atoms were placed at calculated positions; C—H = 0.93 Å for aromatic H, C—H = 0.96 Å for methoxyl H. They were refined using a riding model with *U*_iso_(H) = 1.2 *U*_eq_ (C) and *U*_iso_(H) =1.5 *U*_eq_ (C), respectively.

### Crystal data

**Table:**

C_11_H_10_ClNO_2_	*F*(000) = 464
*M**_r_* = 223.65	*D*_x_ = 1.447 Mg m^−^^3^
Monoclinic, *P*2_1_/*c*	Mo *K*α radiation, λ = 0.71073 Å
*a* = 12.5530 (17) Å	Cell parameters from 30 reflections
*b* = 4.6499 (7) Å	θ = 3–25°
*c* = 18.274 (3) Å	µ = 0.35 mm^−^^1^
β = 105.786 (2)°	*T* = 296 K
*V* = 1026.4 (3) Å^3^	Block, pink
*Z* = 4	0.3 × 0.2 × 0.2 mm

### Data collection

**Table:**

Rigaku SCXmini diffractometer	*R*_int_ = 0.034
Radiation source: fine-focus sealed tube	θ_max_ = 25.0°, θ_min_ = 1.7°
graphite	*h* = −14→14
ω scans	*k* = −5→5
6840 measured reflections	*l* = −21→21
1808 independent reflections	3 standard reflections every 150 reflections
1542 reflections with *I* > 2σ(*I*)	intensity decay: none

### Refinement

**Table:**

Refinement on *F*^2^	Secondary atom site location: difference Fourier map
Least-squares matrix: full	Hydrogen site location: inferred from neighbouring sites
*R*[*F*^2^ > 2σ(*F*^2^)] = 0.037	H-atom parameters constrained
*wR*(*F*^2^) = 0.115	*w* = 1/[σ^2^(*F*_o_^2^) + (0.0662*P*)^2^ + 0.1562*P*] where *P* = (*F*_o_^2^ + 2*F*_c_^2^)/3
*S* = 1.08	(Δ/σ)_max_ < 0.001
1808 reflections	Δρ_max_ = 0.29 e Å^−^^3^
139 parameters	Δρ_min_ = −0.22 e Å^−^^3^
1 restraint	Extinction correction: *SHELXL97* (Sheldrick, 2008), Fc^*^=kFc[1+0.001xFc^2^λ^3^/sin(2θ)]^-1/4^
Primary atom site location: structure-invariant direct methods	Extinction coefficient: 0.029 (4)

### Special details

**Table:**

Geometry. All e.s.d.'s (except the e.s.d. in the dihedral angle between two l.s. planes) are estimated using the full covariance matrix. The cell e.s.d.'s are taken into account individually in the estimation of e.s.d.'s in distances, angles and torsion angles; correlations between e.s.d.'s in cell parameters are only used when they are defined by crystal symmetry. An approximate (isotropic) treatment of cell e.s.d.'s is used for estimating e.s.d.'s involving l.s. planes.
Refinement. Refinement of *F*^2^ against ALL reflections. The weighted *R*-factor *wR* and goodness of fit *S* are based on *F*^2^, conventional *R*-factors *R* are based on *F*, with *F* set to zero for negative *F*^2^. The threshold expression of *F*^2^ > σ(*F*^2^) is used only for calculating *R*-factors(gt) *etc*. and is not relevant to the choice of reflections for refinement. *R*-factors based on *F*^2^ are statistically about twice as large as those based on *F*, and *R*- factors based on ALL data will be even larger.

### Fractional atomic coordinates and isotropic or equivalent isotropic displacement parameters (Å^2^)

**Table:**

	*x*	*y*	*z*	*U*_iso_*/*U*_eq_	
C1	0.18445 (16)	0.9064 (4)	0.34245 (10)	0.0508 (5)	
C2	0.23248 (18)	0.7745 (5)	0.40978 (11)	0.0608 (5)	
H2	0.2071	0.8077	0.4523	0.073*	
C3	0.32020 (19)	0.5895 (5)	0.41333 (12)	0.0668 (6)	
H3	0.3522	0.4999	0.4596	0.080*	
C4	0.31375 (16)	0.6656 (4)	0.28823 (10)	0.0486 (5)	
C5	0.35750 (15)	0.6077 (4)	0.22615 (11)	0.0503 (5)	
H5	0.4173	0.4832	0.2326	0.060*	
C6	0.31298 (14)	0.7325 (4)	0.15691 (10)	0.0469 (4)	
C7	0.22217 (14)	0.9277 (4)	0.14727 (9)	0.0443 (4)	
C8	0.17947 (14)	0.9890 (4)	0.20641 (9)	0.0441 (4)	
H8	0.1208	1.1173	0.1997	0.053*	
C9	0.22416 (14)	0.8581 (3)	0.27829 (9)	0.0437 (4)	
C10	0.43422 (17)	0.4878 (5)	0.09810 (13)	0.0659 (6)	
H10A	0.4997	0.5563	0.1344	0.099*	
H10B	0.4482	0.4703	0.0492	0.099*	
H10C	0.4142	0.3033	0.1139	0.099*	
C11	0.09705 (17)	1.2379 (5)	0.06201 (12)	0.0580 (5)	
H11A	0.0339	1.1499	0.0729	0.087*	
H11B	0.0780	1.2972	0.0097	0.087*	
H11C	0.1195	1.4025	0.0942	0.087*	
Cl1	0.07193 (4)	1.13418 (12)	0.33477 (3)	0.0656 (3)	
N1	0.36194 (14)	0.5307 (4)	0.35576 (9)	0.0623 (5)	
O1	0.34547 (11)	0.6869 (3)	0.09331 (7)	0.0585 (4)	
O2	0.18526 (11)	1.0371 (3)	0.07552 (7)	0.0561 (4)	

### Atomic displacement parameters (Å^2^)

**Table:**

	*U*^11^	*U*^22^	*U*^33^	*U*^12^	*U*^13^	*U*^23^
C1	0.0588 (11)	0.0528 (10)	0.0423 (10)	−0.0142 (9)	0.0164 (8)	−0.0043 (8)
C2	0.0742 (13)	0.0699 (12)	0.0404 (11)	−0.0112 (10)	0.0193 (10)	0.0013 (9)
C3	0.0824 (14)	0.0736 (13)	0.0398 (11)	−0.0076 (11)	0.0088 (10)	0.0115 (10)
C4	0.0543 (10)	0.0463 (10)	0.0428 (10)	−0.0079 (8)	0.0094 (8)	0.0014 (8)
C5	0.0505 (10)	0.0487 (10)	0.0506 (11)	0.0030 (8)	0.0120 (8)	0.0001 (8)
C6	0.0498 (10)	0.0491 (9)	0.0438 (10)	−0.0047 (8)	0.0162 (8)	−0.0054 (8)
C7	0.0504 (10)	0.0460 (9)	0.0363 (9)	−0.0045 (7)	0.0114 (7)	−0.0014 (7)
C8	0.0467 (9)	0.0452 (9)	0.0405 (9)	−0.0029 (7)	0.0121 (7)	−0.0012 (7)
C9	0.0487 (9)	0.0445 (9)	0.0381 (9)	−0.0113 (7)	0.0123 (7)	−0.0036 (7)
C10	0.0635 (12)	0.0696 (13)	0.0717 (14)	0.0061 (11)	0.0303 (10)	−0.0091 (11)
C11	0.0643 (12)	0.0611 (11)	0.0474 (11)	0.0071 (10)	0.0134 (9)	0.0054 (9)
Cl1	0.0735 (4)	0.0778 (4)	0.0532 (4)	0.0040 (3)	0.0302 (3)	−0.0051 (2)
N1	0.0685 (11)	0.0630 (10)	0.0506 (10)	−0.0002 (8)	0.0083 (8)	0.0112 (8)
O1	0.0633 (8)	0.0695 (9)	0.0478 (8)	0.0123 (7)	0.0239 (6)	−0.0024 (6)
O2	0.0675 (8)	0.0654 (8)	0.0380 (7)	0.0129 (7)	0.0188 (6)	0.0068 (6)

### Geometric parameters (Å, °)

**Table:**

C1—C2	1.360 (3)	C6—C7	1.430 (3)
C1—C9	1.411 (3)	C7—C8	1.361 (2)
C1—Cl1	1.740 (2)	C7—O2	1.365 (2)
C2—C3	1.385 (3)	C8—C9	1.418 (2)
C2—H2	0.9300	C8—H8	0.9300
C3—N1	1.325 (3)	C10—O1	1.433 (2)
C3—H3	0.9300	C10—H10A	0.9600
C4—N1	1.370 (2)	C10—H10B	0.9600
C4—C9	1.410 (3)	C10—H10C	0.9600
C4—C5	1.414 (3)	C11—O2	1.418 (2)
C5—C6	1.366 (3)	C11—H11A	0.9600
C5—H5	0.9300	C11—H11B	0.9600
C6—O1	1.349 (2)	C11—H11C	0.9600
			
C2—C1—C9	120.67 (19)	C7—C8—C9	120.25 (17)
C2—C1—Cl1	119.85 (16)	C7—C8—H8	119.9
C9—C1—Cl1	119.48 (14)	C9—C8—H8	119.9
C1—C2—C3	118.27 (19)	C4—C9—C1	116.33 (16)
C1—C2—H2	120.9	C4—C9—C8	119.53 (16)
C3—C2—H2	120.9	C1—C9—C8	124.14 (17)
N1—C3—C2	124.90 (18)	O1—C10—H10A	109.5
N1—C3—H3	117.6	O1—C10—H10B	109.5
C2—C3—H3	117.6	H10A—C10—H10B	109.5
N1—C4—C9	123.24 (18)	O1—C10—H10C	109.5
N1—C4—C5	117.57 (17)	H10A—C10—H10C	109.5
C9—C4—C5	119.19 (16)	H10B—C10—H10C	109.5
C6—C5—C4	120.80 (17)	O2—C11—H11A	109.5
C6—C5—H5	119.6	O2—C11—H11B	109.5
C4—C5—H5	119.6	H11A—C11—H11B	109.5
O1—C6—C5	126.01 (17)	O2—C11—H11C	109.5
O1—C6—C7	114.29 (15)	H11A—C11—H11C	109.5
C5—C6—C7	119.69 (17)	H11B—C11—H11C	109.5
C8—C7—O2	125.52 (16)	C3—N1—C4	116.59 (18)
C8—C7—C6	120.54 (15)	C6—O1—C10	117.47 (15)
O2—C7—C6	113.94 (15)	C7—O2—C11	117.24 (14)

### Hydrogen-bond geometry (Å, °)

**Table:**

*D*—H···*A*	*D*—H	H···*A*	*D*···*A*	*D*—H···*A*
C8—H8···Cl1	0.93	2.70	3.0827 (17)	105
